# Effect of tube current on computed tomography radiomic features

**DOI:** 10.1038/s41598-018-20713-6

**Published:** 2018-02-05

**Authors:** Dennis Mackin, Rachel Ger, Cristina Dodge, Xenia Fave, Pai-Chun Chi, Lifei Zhang, Jinzhong Yang, Steve Bache, Charles Dodge, A. Kyle Jones, Laurence Court

**Affiliations:** 1 Department of Radiation Physics, The University of Texas MD Anderson Cancer Center, Houston, TX 77030 USA; 2 Graduate School of Biomedical Sciences, The University of Texas Health Science Center at Houston, Houston, TX 77030 USA; 3 Department of Radiology, Texas Children’s Hospital, Houston, TX 77030 USA; 4 Department of Imaging Physics, The University of Texas MD Anderson Cancer Center, Houston, TX 77030 USA; 5 Imaging Physics, Houston Methodist Hospital, Houston, TX 77030 USA

## Abstract

Variability in the x-ray tube current used in computed tomography may affect quantitative features extracted from the images. To investigate these effects, we scanned the Credence Cartridge Radiomics phantom 12 times, varying the tube current from 25 to 300 mA∙s while keeping the other acquisition parameters constant. For each of the scans, we extracted 48 radiomic features from the categories of intensity histogram (n = 10), gray-level run length matrix (n = 11), gray-level co-occurrence matrix (n = 22), and neighborhood gray tone difference matrix (n = 5). To gauge the size of the tube current effects, we scaled the features by the coefficient of variation of the corresponding features extracted from images of non-small cell lung cancer tumors. Variations in the tube current had more effect on features extracted from homogeneous materials (acrylic, sycamore wood) than from materials with more tissue-like textures (cork, rubber particles). Thirty-eight of the 48 features extracted from acrylic were affected by current reductions compared with only 2 of the 48 features extracted from rubber particles. These results indicate that variable x-ray tube current is unlikely to have a large effect on radiomic features extracted from computed tomography images of textured objects such as tumors.

## Introduction

The field of radiomics, in which quantitative image features are used to determine the tumor phenotype, has been demonstrated to have various potential roles in clinical decision-making. These include classifying tumors (e.g., benign or malignant), determining mutation status, improving patient risk stratification, predicting appropriate treatment strategies, and monitoring treatment response to improve outcome predictions^1–9^. However, radiomic features and results are sensitive to a variety of noise sources. For example, inter-scanner variations in image features can be relatively large^9,10^. Similarly, details of the imaging protocol, such as pixel size, can significantly affect the values of the calculated features^11^.

To maximize the amount of useful information obtained from computed tomography (CT) images in radiomics (and avoid incorrect interpretation of results), researchers must understand all sources of noise. This understanding can help with the development of solutions, such as image preprocessing, to mitigate the effects of the noise. In retrospective studies, understanding of the noise sources could be used to guide which image data are analyzed (e.g., only images with a pixel size within a specified range). In prospective studies, noise analysis could be used to guide the creation of harmonized imaging protocols in which the most important parameters are controlled. Examples of noise sources that have been examined in previous work include different CT scanners, pixel size, image spacing, and reconstruction kernels^10,12–15^.

The impact of tube current on diagnostic tasks has been covered extensively in the literature^16–21^, but only preliminary data are available regarding the effects of tube current in radiomics studies. Fave *et al*. simulated the effect of tube current on measured image features by adding Gaussian noise to patient CT images^22^. They observed no significant effect on image features (compared with inter-patient variations) but acknowledged that their noise model was very basic and did not properly reflect the changes in noise as the tube current was reduced. Larue *et al*. found that optimizing the number of gray-levels in images to improve prognostic value did not adversely affect feature stability. Further, they found that feature values were not correlated to tube currents or to slice thickness after resampling^23^. A study from Mahmood *et al*. raised questions about the robustness of image texture features. They imaged an anthropomorphic phantom on scanners from three manufacturers, and no features had CCC values greater than 0.9^24^. A more reliable answer would involve scanning an object using different tube currents and then assessing the impact of this variation on the calculated image features.

Mackin *et al*. recently described a texture phantom that can be used to assess the impact of the imaging device or protocol on extracted image features^10^. In the current study, we experimentally examined the effects of tube current on quantitative image features by scanning this texture phantom using a range of tube current values.

## Methods and Materials

**Figure 1:**
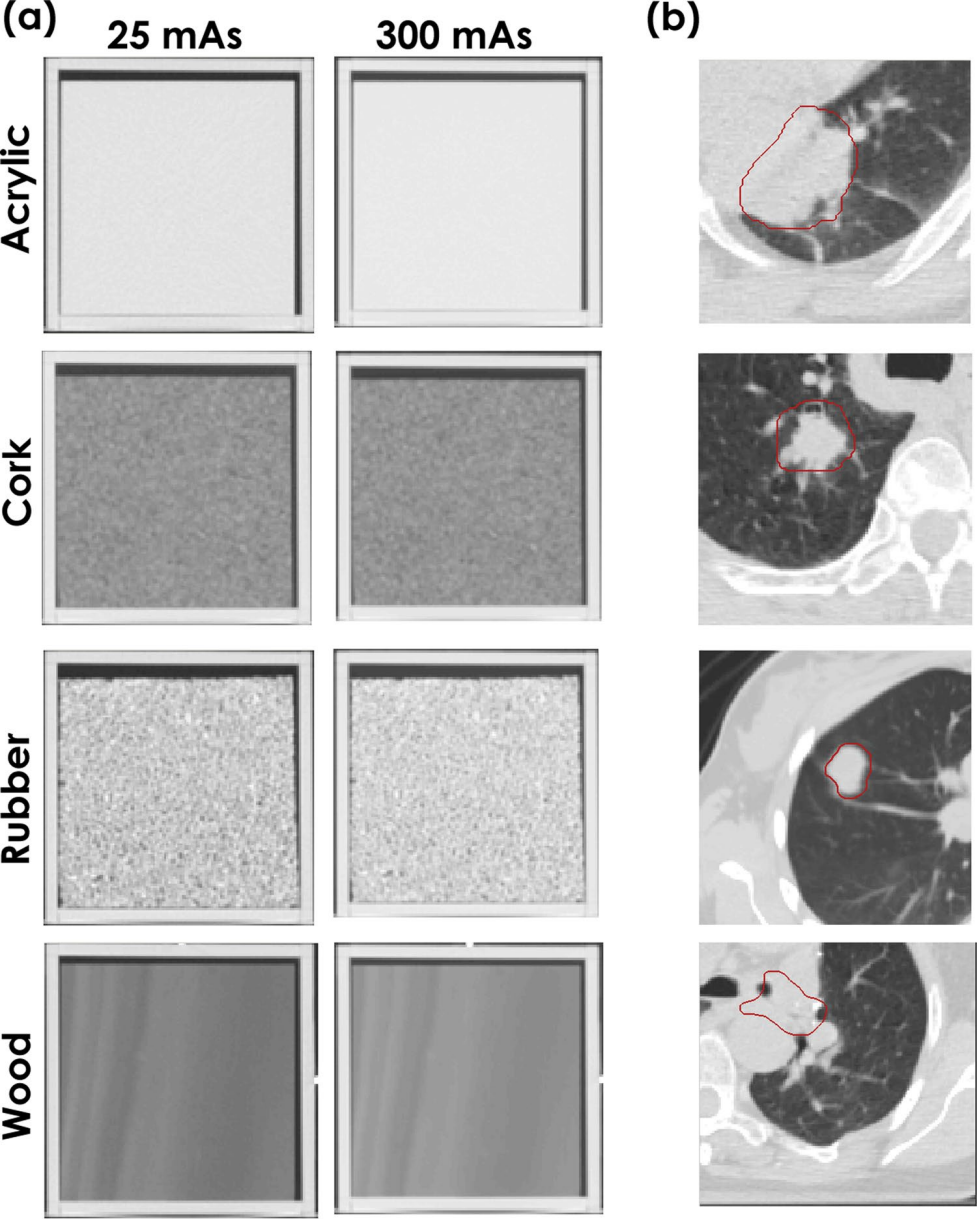
Cross sections of the (**a**) Credence Cartridge Radiomics phantom and (**b**) a representative cross sections of a non-small cell lung cancer tumors. The window level was (1600, −300) for all images. The columns in (**a**) show images acquired using tube currents of 25 and 300 mA∙s. The images in (**b**) were acquired with tube currents of 100 or 200 mA∙s.

The credence cartridge radiomics (CCR) phantom as described by Mackin *et al*.^10^ was used to study the effects of tube current on radiomic features. The CCR phantom has ten cartridges with various textures. In the current study, we analyzed four of the cartridges: solid acrylic, cork, rubber particles, and wood. These were selected to give a full range of textures from minimal to highly varied, similar to the texture of non-small cell lung cancer tumors (Fig. 1).

The phantom was imaged on a GE LightSpeed VCT scanner (GE Healthcare, Waukesha, WI) and a Toshiba Aquilion ONE scanner (Toshiba Medical Systems, Tustin, CA) using a range of tube current settings. The GE LightSpeed images were acquired in helical mode at 120 kVp, 0.969 pitch, STANDARD reconstruction kernel, 50-cm display field of view, and 2.5-mm image thickness. Each voxel was 0.98 × 0.98 × 2.5 mm^3^. Twelve scans were acquired at 25, 50, 75, 100, 125, 150, 175, 200, 225, 250, 275, and 300 mA∙s. The Toshiba Aquilion ONE scans were acquired in helical mode at 120 kVp, 1.4 pitch, FC18 reconstruction kernel, 50-cm display field of view, and 5-mm image thickness. Each voxel was 0.98 × 0.98 × 5.0 mm^3^. Ten scans were acquired at 25, 50, 75, 100, 125, 150, 175, 200, 225, and 250 mA∙s.

The images were imported into IBEX, a freely available radiomics software program^25^. The location of a small radiopaque marker on the edge of the phantom was manually identified, and its image coordinates were entered into an in-house Python script (Python version 2.7), which created a rectangular region of interest (ROI) of 8 × 8 × 2 cm^3^ for each cartridge in each scan. The same ROI file was used for all images from a particular scanner. Results for smaller (2 × 2 × 2 cm^3^) voxels are included in the supplementary materials. Forty-eight features were calculated in IBEX: 10 intensity histogram, 22 gray-level co-occurrence matrix^26^, 11 gray-level run length matrix^27,28^, and 5 neighborhood gray tone difference matrix features^29^ (Table 1). These features were selected because they are commonly used in radiomics studies^30,31^. As noted below, features were calculated with one and four CT numbers per bin. The texture features were calculated for each slice in the ROIs and then combined, a procedure referred to as 2.5D^25^.

**Table 1:** Features extracted for analysis.

Intensity histogram	Gray-level co-occurrence matrix	Gray-level run length matrix	Neighborhood gray tone difference matrix
Energy	Auto Correlation	Gray Level Nonuniformity	Busyness
Entropy	Cluster Prominence	High Gray Level Run Emphasis	Coarseness
Kurtosis	Cluster Shade	Long Run Emphasis	Complexity
Local entropy max	Cluster Tendendcy	Long Run High Gray Level Emphasis	Contrast
Local entropy mean	Contrast	Long Run Low Gray Level Emphasis	Texture Strength
Mean	Correlation	Low Gray Level Run Emphasis	
Median	Difference Entropy	Run Length Nonuniformity	
Range	Dissimilarity	Run Percentage	
Skewness	Energy	Short Run Emphasis	
Standard deviation	Entropy	Short Run High Gray Level Emphasis	
Uniformity	Homogeneity	Short Run Low Gray Level Emphasis	
	Homogeneity 2		
	Information Measure Correlation 1		
	Information Measure Correlation 2		
	Inverse Diff Moment Norm		
	Inverse Diff Norm		
	Inverse Variance		
	Maximum Probability		
	Sum Average		
	Sum Entropy		
	Sum Variance		
	Variance		

Reducing the tube current used in CT scans will increase the noise in the images. In this study our primary concern is not measure the size of tube current effect in the phantom materials. Our primary concern is to gauge the size of the effect tube current may have on radiomics studies of patients. Therefore, we used a metric that scales the effect seen in phantom materials by the variability in patients. If a feature is highly variable in patients, a small tube current effect is unlikely to weaken a radiomic feature. On the other hand, a large tube current effect is likely to weaken a radioimic feature when the patient variability is small. To gauge the size of the effects of variable tube current relative to the variability in patients, we normalized the extracted feature values by the coefficient of variation for the same features extracted from CT images of non-small cell lung cancer tumors. This patient-normalized feature, ${\hat{f}}_{i}$, was defined as

1 $${\hat{f}}_{i}=\frac{({f}_{i}-{f}_{0})/{f}_{0}}{{\sigma }_{T,i}/{\mu }_{T,i}}\,$$

where *f*_*i*_ is the feature value for a given tube current value *i* (mA∙s) and *f*_0_ is the feature value at the baseline tube current value (300 mA∙s for the GE scanner and 250 mA∙s for the Toshiba scanner). σ_*T*,*i*_ and µ_*T*,*i*_ are the standard deviation and mean, respectively, of the features from the non-small cell lung cancer tumors. The numerator of $\hat{{f}_{i}}$ is the fractional difference for a tube current scan and the baseline, and the denominator is the coefficient of variation for the same feature calculated on 106 non-small cell lung cancer tumors. This normalization assesses the variability caused by tube current relative to inter-patient differences.

The patients in this normalization cohort were part of a clinical trial approved by the Institutional Review Board. Informed consent for participation in the trial was obtained for all patients, and all procedures were performed in accordance with the Declaration of Helsinki on Ethical Issues. Additional informed consent for this retrospective study was waved by the Institutional Review Board. The gross tumor volumes from the end-of-exhale phase of the planning CT images were used as the ROIs for feature extraction. The features were extracted after applying a threshold of 900 HU. The end-of-exhale phase is considered the most stable^22,32,33^. The mean and median tumor ROI volumes were 96 and 42 cm^3^ respectively (range 5–568 cm^3^). ROIs smaller than 5 cm^3^ were excluded from the normalization cohort. This patient cohort was part of a prior radiomics study which details its clinical characteristics^9^.

Features from the phantom and patients were extracted in four ways: (1) no preprocessing, (2) intensity rescaling (10-bit depth rescaling), (3) Butterworth smoothing, and (4) Butterworth smoothing and intensity rescaling. These preprocessing techniques were chosen on the basis of work by Fave *et al*., who showed that preprocessing affects the significance of features in prognostic models^34^. Rescaling the intensity from the initial 4096 bins (12-bit) to 1024 bins (10-bit) combines 4 CT numbers per bin.

### Data availability

The datasets generated during and/or analyzed during the current study are available from the corresponding author on reasonable request.

## Results

**Figure 2:**
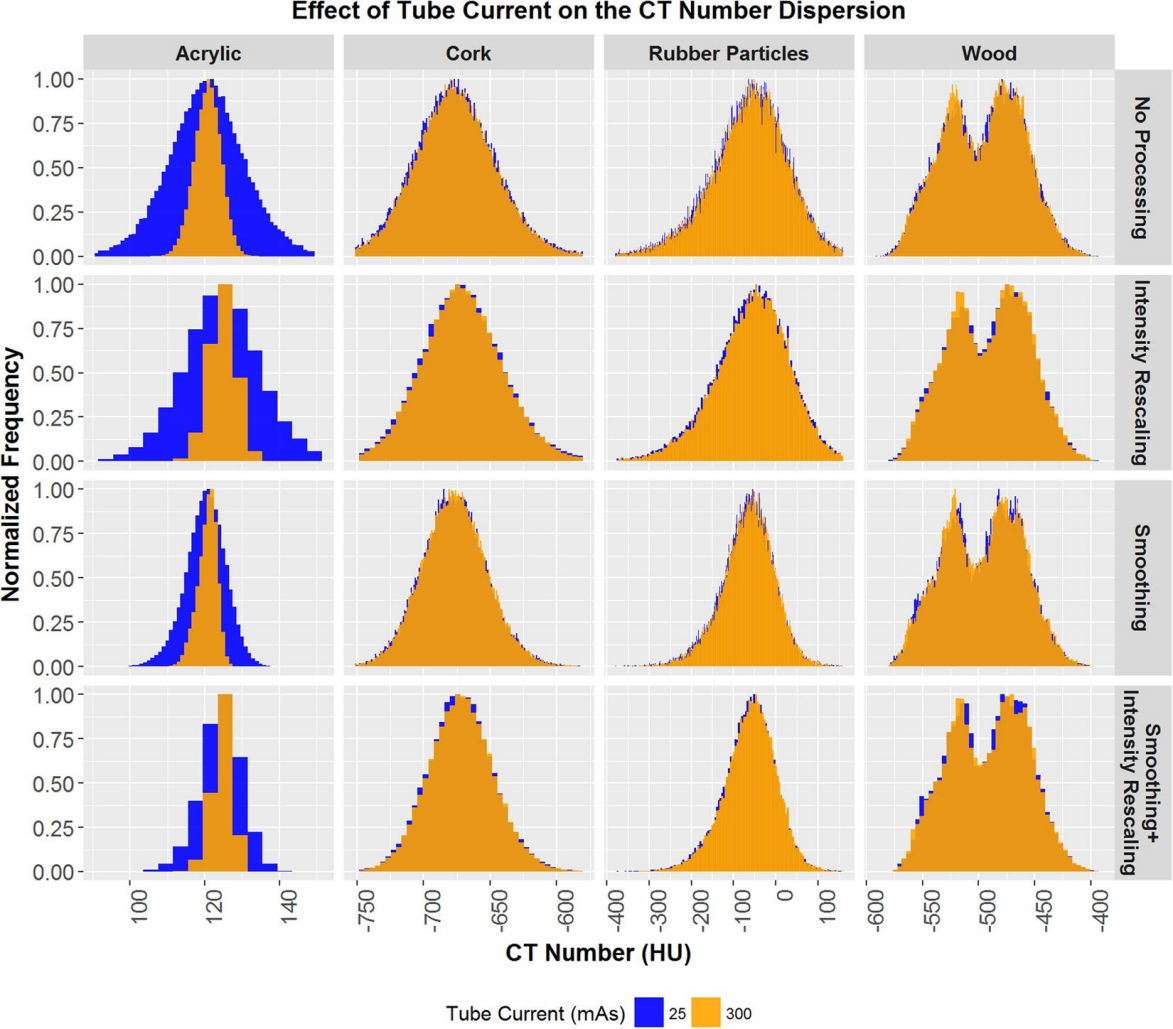
Image intensity histograms for the acrylic, cork, rubber particle, and sycamore wood cartridges acquired using 25 and 300 mA∙s tube current on a GE LightSpeed VCT computed tomography (CT) scanner. For display purposes, the frequency values have been rescaled so that the maximum is 1.0.

The effects of tube current on image intensity histograms for the acrylic, cork, rubber particle, and sycamore wood cartridges from images acquired on the GE scanner are shown in Fig. 2 (results for images acquired on the Toshiba scanner are shown in Supplementary Fig. 1). Results did not vary substantially between the two scanners used. Therefore, all figures show results obtained using the GE LightSpeed VCT scanner, and results obtained using the Toshiba Aquilion ONE scanner are shown in corresponding supplementary figures. The acrylic cartridge image from the 25 mA∙s scan had a much greater dispersion of intensities than did the image from the 300 mA∙s scan. The differences between the intensity histograms for these two scans were not as apparent in the images of the other three materials. Preprocessing the images by rescaling, smoothing, or both rescaling and smoothing had only a marginal effect on the dispersion differences.

**Figure 3:**
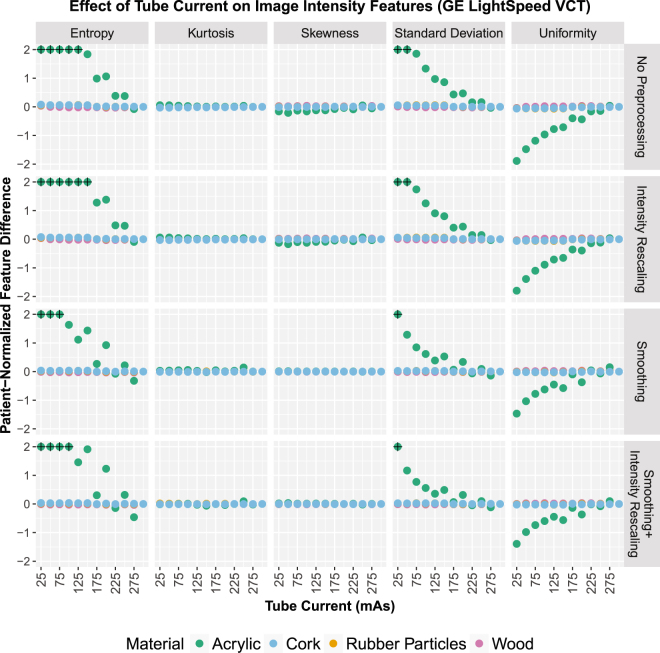
Effects of reduced tube current values on the patient-normalized image intensity feature values for four phantom materials of varying degrees of texture. The effects were larger in materials with less texture, acrylic and wood. The images were acquired using a GE LightSpeed VCT scanner.

The effects of tube current on the intensity features, shown in Fig. 3 for the GE scanner (results for the Toshiba scanner are shown in Supplementary Fig. 2), are consistent with the effects seen in the intensity histograms. The values used in this figure were scaled according to Equation 1 to produce patient-normalized feature differences. Reducing the tube current values produced the largest changes in the acrylic cartridge features. As with the intensity histograms, rescaling the intensities, smoothing the images, or doing both had little effect on the patient-normalized feature values.

**Figure 4:**
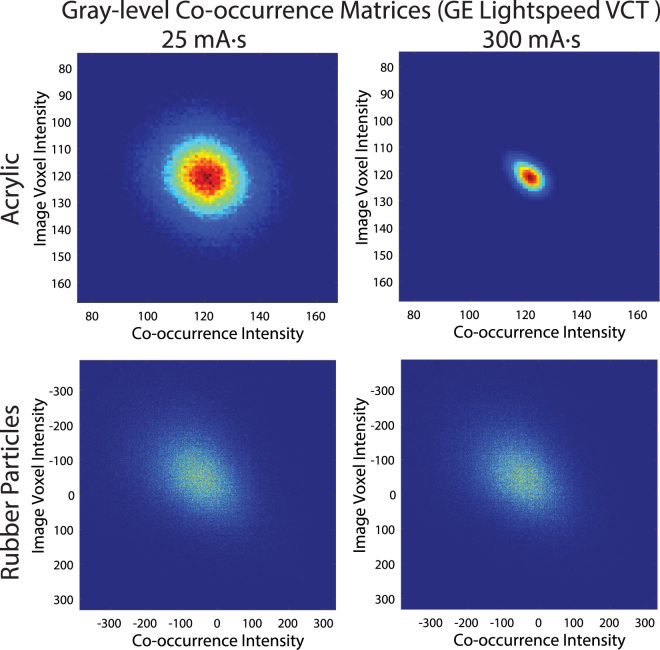
Images of the gray-level co-occurrence matrices for the acrylic and rubber particle cartridges for computed tomography scans acquired using 25 and 300 mA∙s tube current on a GE LightSpeed VCT scanner. For each co-occurrence matrix, the relative frequency of intensity pairs is plotted and scaled from 0 in dark blue to the max value for that matrix in dark red. Differences in the matrices were apparent for the homogeneous acrylic cartridge where increasing the mAs led to a diagonal distribution of intensity pairs compared to the lower mAs with its circular distribution.

**Figure 5:**
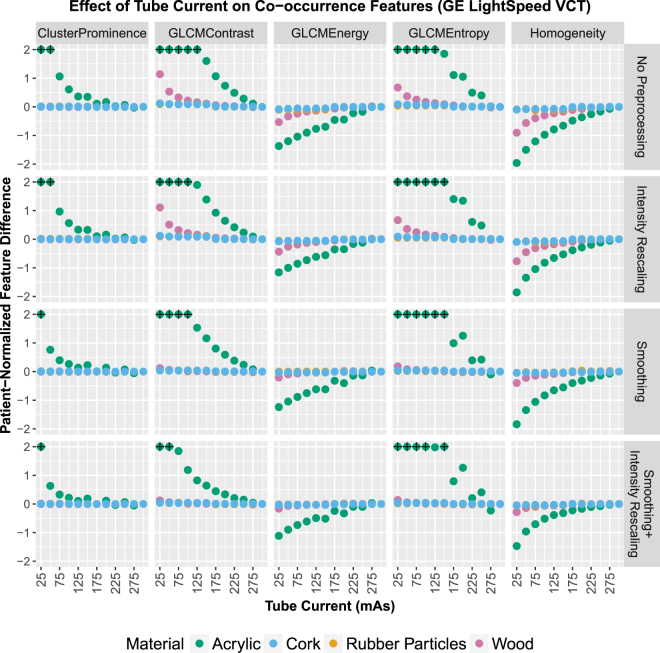
Effects of reduced tube current on radiomic feature values for four phantom materials of varying degrees of texture, obtained using a GE LightSpeed VCT scanner. The effects were larger in materials with less texture, acrylic and sycamore wood. For display purposes, the values are restricted to the range (−2, 2), and points that fall outside the range are marked with a black + symbol.

Grey-level co-occurrence matrices for the acrylic and rubber particles cartridges for images acquired on the GE scanner are shown as images in Fig. 4 (results for images acquired on the Toshiba scanner are shown in Supplementary Fig. 3). Gray-level co-occurrence matrices record the frequency of image intensity values being adjacent each other, or “co-occurring”, in the image. The difference in the dispersion of the CT numbers for the 25 and 300 mA∙s scans produced obvious changes in the gray-level co-occurrence matrix for the acrylic cartridge. This dependence is also evident in the features derived from the gray-level co-occurrence matrices, shown in Fig. 5 (results for images acquired on the Toshiba scanner are shown in Supplementary Fig. 4). The tube current dependence was greatest for the acrylic cartridge. A more moderate effect was seen in the sycamore wood cartridge and little effect was seen in the heterogeneous cork and rubber particles cartridges.

**Figure 6:**
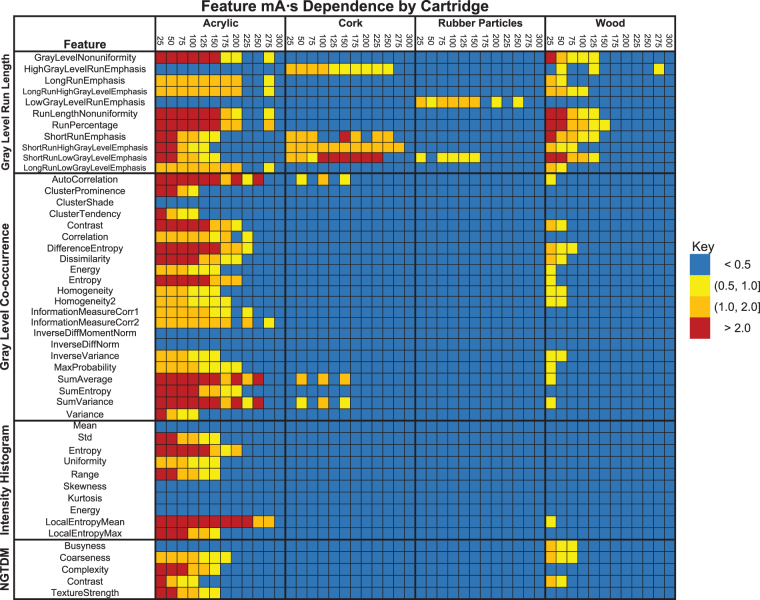
Maps of the patient-normalized features extracted from the acrylic, cork, rubber particle, and sycamore wood cartridges. The columns represent the tube current (mA∙s) used to acquire the computed tomography scan and are grouped by the material. Colors other than blue indicate that the effect of the reduced tube current is large relative to the variability of the feature calculated for tumor samples from patients with non-small cell lung cancer. The almost solid blue table for the rubber particle cartridges indicates that reducing the tube current has little effect on the radiomic features. The images were acquired using a GE LightSpeed VCT scanner. NGTDM, neighborhood gray tone difference matrix.

A high-level look at the 48 features from the four feature groups is shown in Fig. 6, for images acquired on the GE scanner (results for images acquired on the Toshiba scanner are shown in Supplementary Fig. 5). In this figure, the patient-normalized feature values are color-coded into one of four categories indicating the degree to which the feature values depend on the tube current value used when the images were acquired. The features extracted from the most homogeneous materials, acrylic and sycamore wood, were much more dependent on tube current than were the features extracted from the more textured materials, cork and rubber particles. More specifically, 25 of 48 features extracted from the acrylic cartridge were strongly dependent on tube current (${\hat{f}}_{i} > 2$). In contrast, ${\hat{f}}_{i} > 0.5$ was observed for only two of the features extracted from the rubber particles cartridge.

## Discussion

We investigated the effect of reducing tube current on materials with varying amounts of texture and found that objects with more intrinsic texture were not substantially affected by tube current changes. Reducing the tube current of a CT scan increases the image noise, and therefore increases the spread of CT numbers. We found that the impact of this noise was more apparent for homogeneous materials (e.g., acrylic) than for textured materials. The increased spread observed in the intensity histogram for the acrylic cartridge at 25 mA∙s compared with 300 mA∙s implies that image intensity features for this cartridge are expected to be dependent on tube current. For the most textured cartridges, rubber particles and cork, dispersion at the lower tube current values was minimal, and thus, features extracted from these types of materials are not expected to be dependent on tube current.

Indeed, the expected feature dependence was observed: feature dependence in acrylic varied whereas this dependence in rubber particles and cork – which have texture more similar to NSCLC tumors – stayed constant. This is shown through the plots of the patient-normalized feature differences (Figs 3 and 5), in which values for cork and rubber particles stayed near 0 for all points whereas values for acrylic approached 2 or -2 or even extended beyond the displayed range for many features. These findings suggest that when a material has minimal texture, such as acrylic, the texture feature values are heavily dependent on noise, i.e., tube current values. Materials that did have texture showed little effect from tube current variation. Additionally, image preprocessing (intensity rescaling or smoothing) did not substantially change this relationship. Overall, these results are consistent with the simpler model that was examined by Fave *et al*.^22^. The results presented here indicate that retrospective radiomics studies should not be significantly affected by variations in tube current. In other words, there should be no need to exclude patient data from retrospective studies on the basis of differences in tube current alone.

These results also indicate that harmonizing tube current values between scans need not be a big concern when planning prospective radiomics studies. This is important because most scanners now modulate mA∙s to control the overall noise level, and these settings may be determined locally (e.g., on the basis of radiologist feedback). Thus, it may be difficult to harmonize the tube current between institutions. Instead, harmonization can focus on details of the image reconstruction such as pixel size and reconstruction kernel, which can both be achieved in a second reconstruction which is specifically designed for radiomics studies and which does not increase the radiation dose to the patient.

The current study has a few limitations. Phantom materials are not perfect surrogates for tissue, and some quantitative features extracted from some human tissues might be more sensitive to changes in the tube current values. It is possible that some regions in tumors might be more sensitive to the tube current than other regions. In addition, the acquired scans had an image thicknesses of 2.5 or 5.0 mm and all ROIs had a volume of 128 cm^3^. These factors may reduce the impact of tube current owing to the reduced effect of noise. We repeated our study using smaller, 8 cm^3^, voxels, and found that the results for the smaller ROIs were similar for both the GE and Toshiba scanners (supplementary Figs 6 and 7). Also, the results for 2.5 and 5.0 image thicknesses were similar to each other. Additionally, the current study evaluated only the effects of tube current on measured radiomic features within a defined volume. The effects of tube current on physician delineation were not evaluated. With more noise at lower tube current values, it may be more difficult to determine ROI bounds within patients. A change in the contouring would affect the radiomic features measured^35^. Finally, we did not evaluate many of the other parameters of the imaging protocol, such as tube voltage (kV). These should be investigated in future studies.

## Conclusion

To provide noninvasive and relatively inexpensive biomarkers, radiomic studies may rely on images acquired using diagnostic imaging or radiation therapy simulation protocols. Retrospective reconstruction of medical images where the imaging procedure is performed one time but the images are reconstructed multiple times using a radiomics protocol in addition to the standard protocol may help to standardize the images. For a CT scan, however, some parameters, including the pitch, tube voltage, and tube current, cannot be changed for retrospective reconstruction. Most CT scans of adults use 120 kVp. It seems unlikely that the pitch of helical scans will affect quantitative imaging features. Thus, the tube current, which influences both the overall noise in the image and the radiation dose to the patient, is most concerning of these three parameters. Our finding that radiomic features are robust to changes in tube current in CT studies of tumor-like materials indicates that variations in the tube current used while imaging patients is unlikely to weaken the study. It is unlikely that radiomic image features calculated from CT images of textured objects (such as tumors) are significantly affected by x-ray tube current.

## Electronic supplementary material

Supplementary Material
