# Supplementary material for: Effect of tube current on computed tomography radiomic features
Source: Sci Rep. 2018 Feb 5;8:2354. doi: 10.1038/s41598-018-20713-6 (PMC5799381; doi:10.1038/s41598-018-20713-6)
Supplement: Supplementary file 1 — Supplementary Material [file 41598_2018_20713_MOESM1_ESM.pdf]

Effect of tube current on computed tomography radiomic features: Supplemental information

Dennis Mackin[1], Rachel Ger[1,2], Cristina Dodge[3], Xenia Fave[1,2], Pai-Chun Chi[1], Lifei Zhang[1], Jinzhong Yang[1], Steve Bache[4], Charles Dodge[5], A. Kyle Jones[4], Laurence Court[1]

[1]Department of Radiation Physics, The University of Texas MD Anderson Cancer Center, Houston, TX 77030, USA

[2]Graduate School of Biomedical Sciences, The University of Texas Health Science Center at Houston, Houston, TX 77030, USA

[3]Department of Radiology, Texas Children's Hospital, Houston, TX 77030, USA

[4]Department of Imaging Physics, The University of Texas MD Anderson Cancer Center, Houston, TX 77030, USA

[5]Imaging Physics, Houston Methodist Hospital, Houston, TX 77030, USA

Corresponding author: Dennis Mackin, dsmackin@mdanderson.org

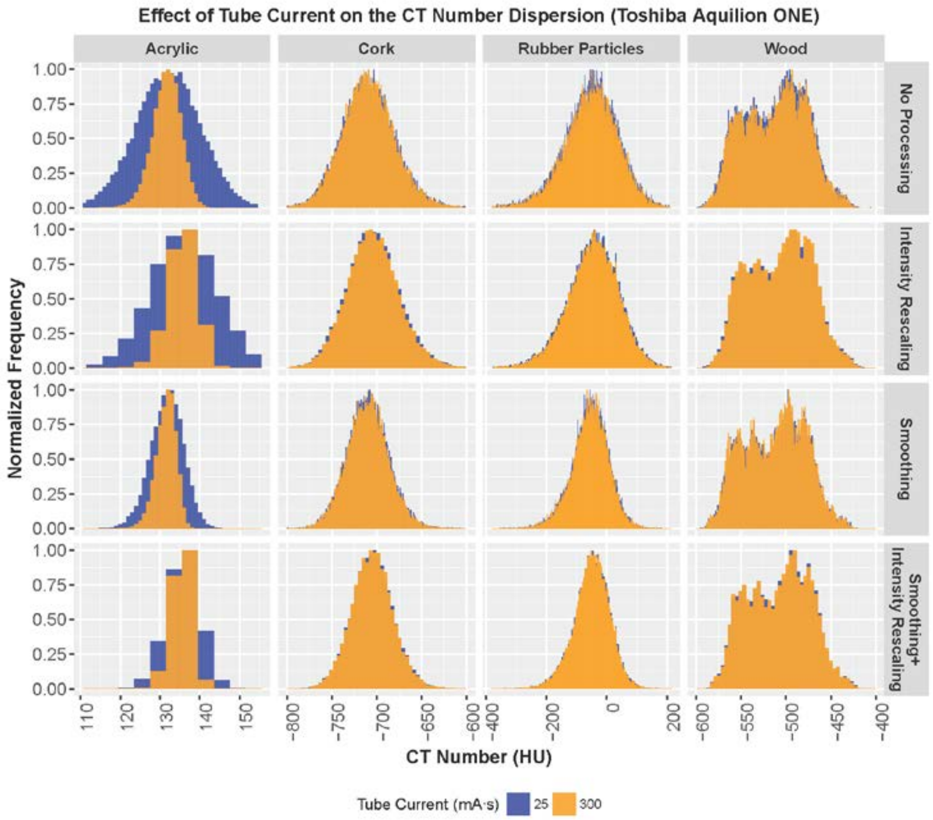

Supplemental Figure 1: Image intensity histograms for the acrylic, cork, rubber particle, and sycamore wood cartridges acquired using 25 and 300 mA·s tube current on a Toshiba Aquilion One scanner. For display purposes, the frequency values have been rescaled so that the maximum is 1.0.

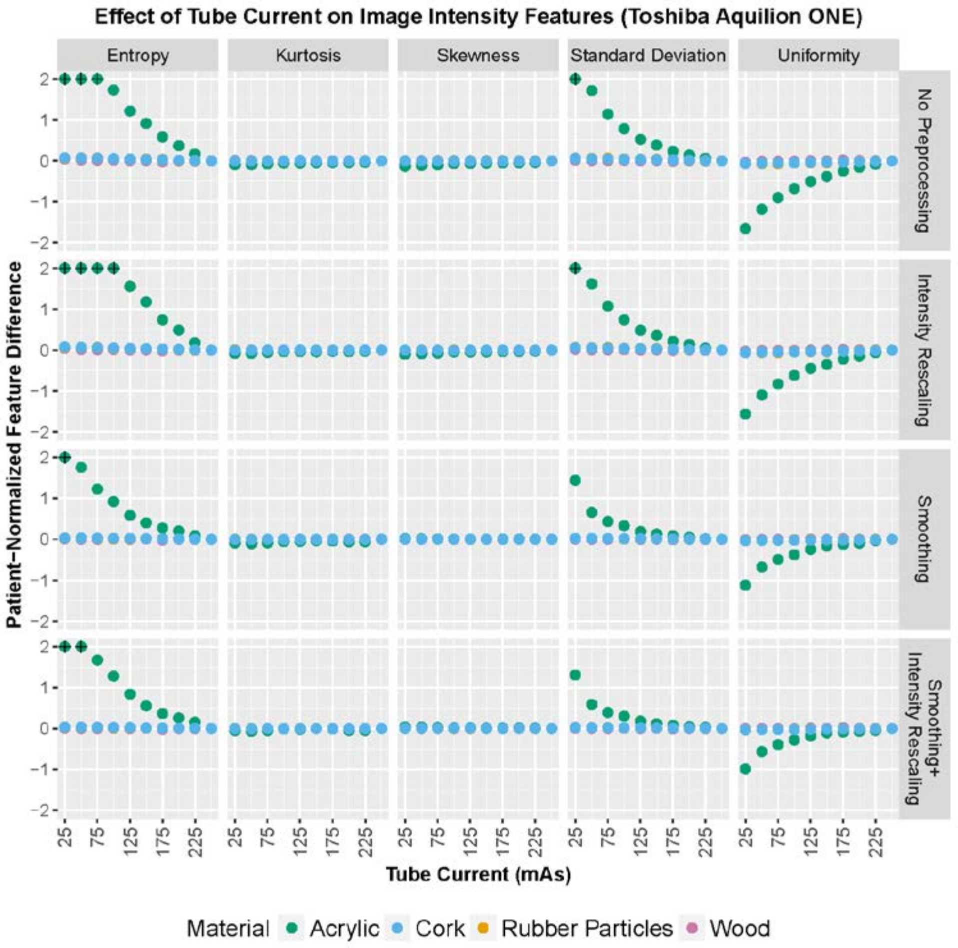

Supplemental Figure 2. Effects of reduced tube current values on the patient-normalized image intensity feature values for four phantom materials of varying degrees of texture. The effects were larger in materials with less texture, acrylic and wood. The images were acquired using a Toshiba Aquilion One scanner.

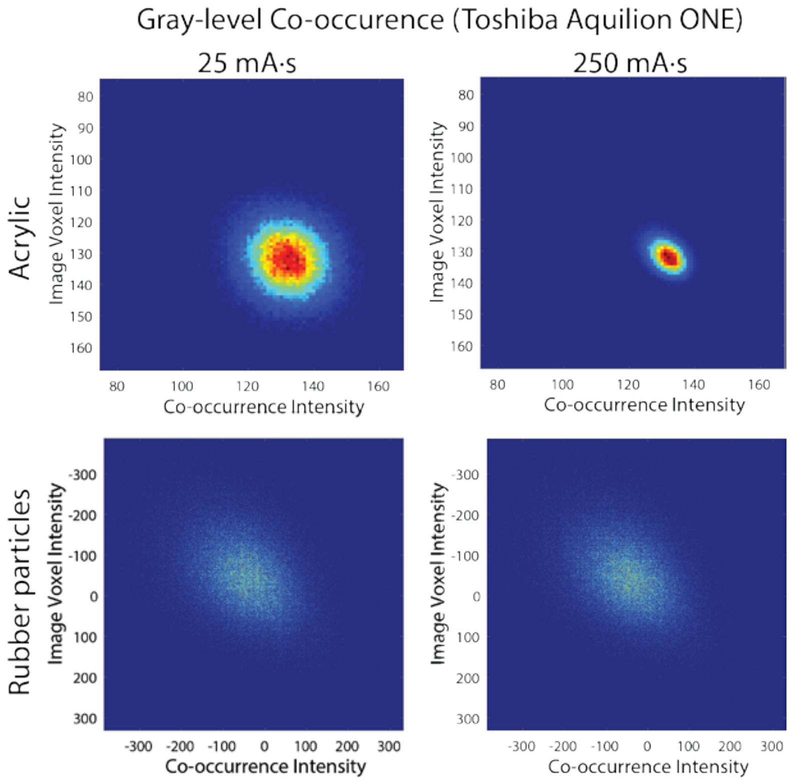

Supplemental Figure 3. Images of the gray-level co-occurrence matrices for the acrylic and rubber particle cartridges for computed tomography scans acquired using 25 and 250 mA·s tube current on a Toshiba Aquilion One scanner. For each co-occurrence matrix, the relative frequency of intensity pairs is plotted and scaled from 0 in dark blue to the max value for that matrix in dark red. Differences in the matrices were apparent for the homogeneous acrylic cartridge where increasing the mAs led to a diagonal distribution of intensity pairs compared to the lower mAs with its circular distribution.

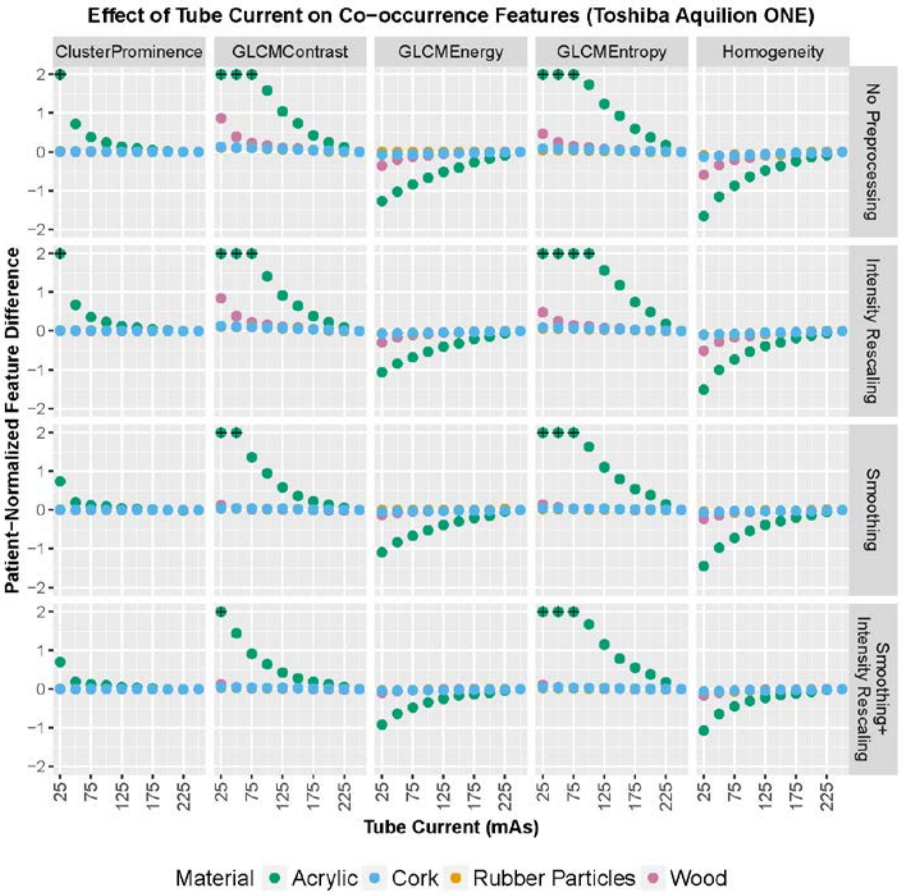

Supplemental Figure 4. Effects of reduced tube current on radiomics feature values for four phantom materials of varying degrees of texture, obtained using a Toshiba Aquilion One scanner. The effects were larger in materials with less texture, acrylic and sycamore wood. For display purposes, the values are restricted to the range (-2, 2), and points that fall outside the range are marked with a black + symbol.

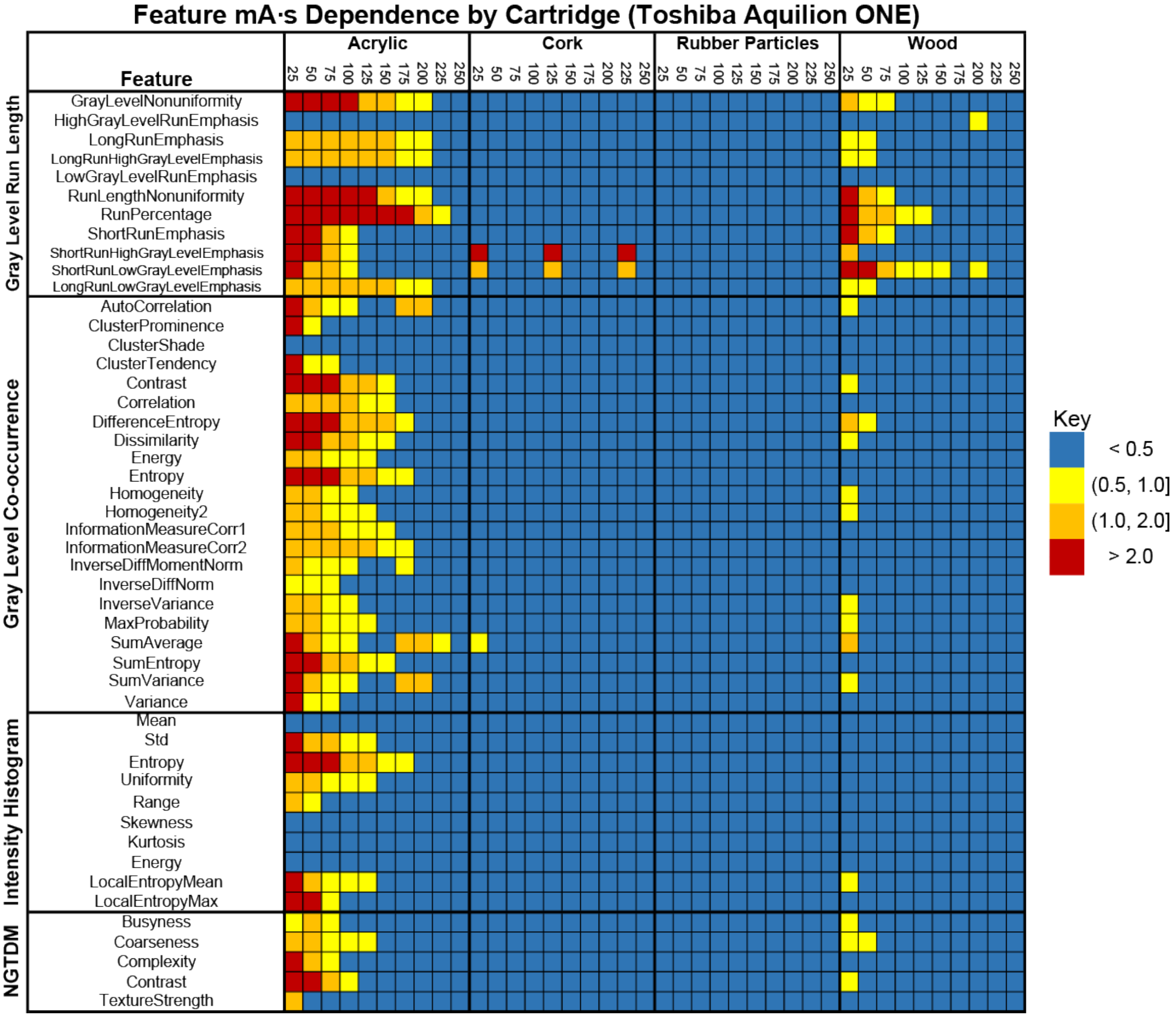

Supplemental Figure 5: Maps of the patient-normalized features extracted from the acrylic, cork, rubber particle, and sycamore wood cartridges. The columns represent the tube current (mA·s) used to acquire the computed tomography scan and are grouped by the material. Colors other than blue indicate that the effect of the reduced tube current is large relative to the variability of the feature calculated for tumor samples from patients with non-small cell lung cancer. The almost solid blue table for the rubber particle cartridges indicates that reducing the tube current has little effect on the radiomics features. The images were acquired using a Toshiba Aquilion One scanner. GLRL indicates gray-level run length; NGTDM, neighborhood gray tone difference matrix.

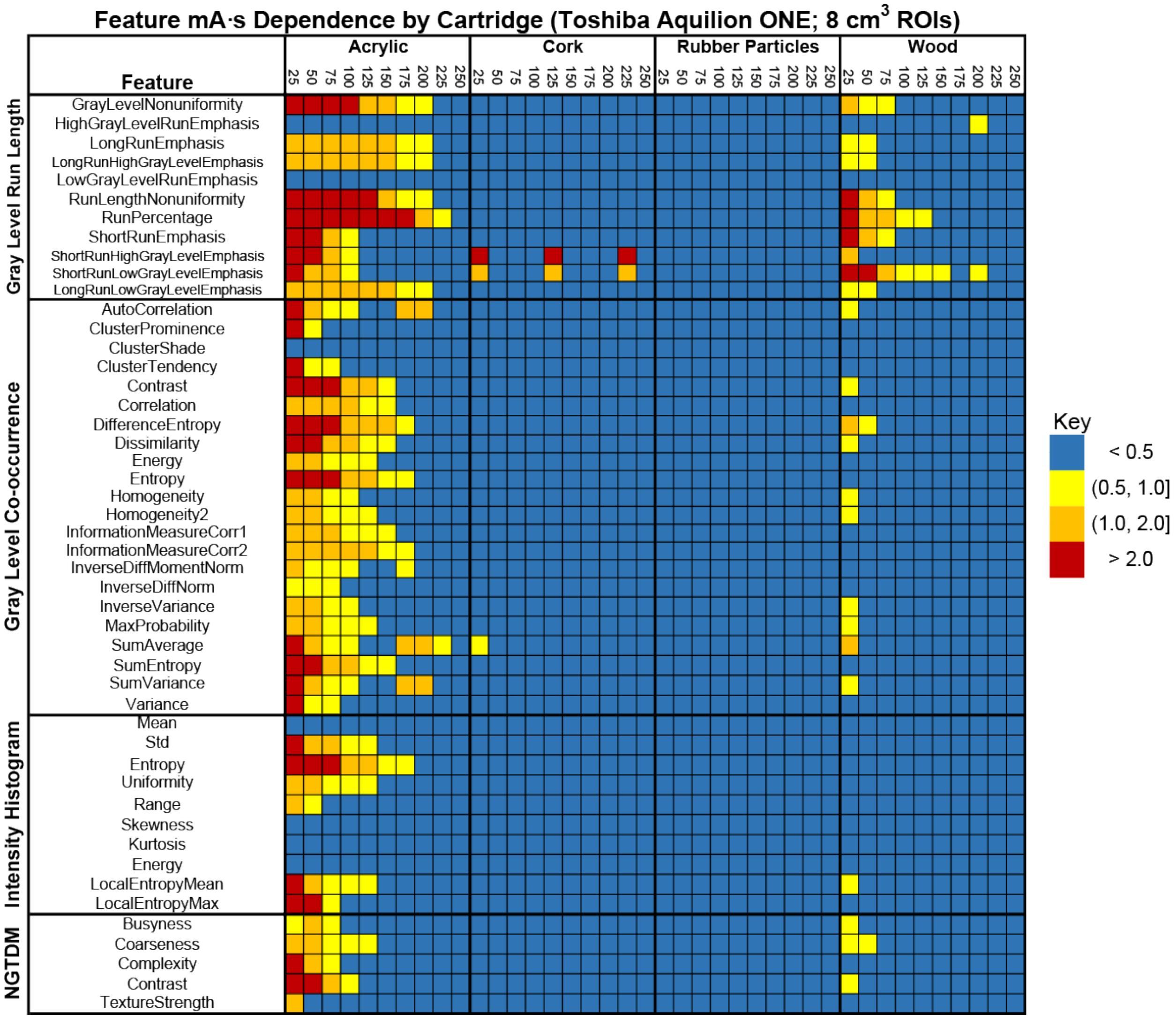

Supplemental Figure 6: Maps of the patient-normalized features extracted from 8 cm^3 ROIs in the acrylic, cork, rubber particle, and sycamore wood cartridges. The columns represent the tube current (mA·s) used to acquire the computed tomography scan and are grouped by the material. Colors other than blue indicate that the effect of the reduced tube current is large relative to the variability of the feature calculated for tumor samples from patients with non-small cell lung cancer. The almost solid blue table for the rubber particle cartridges indicates that reducing the tube current has little effect on the radiomics features. The images were acquired using a Toshiba Aquilion One scanner. GLRL indicates gray-level run length; NGTDM, neighborhood gray tone difference matrix.

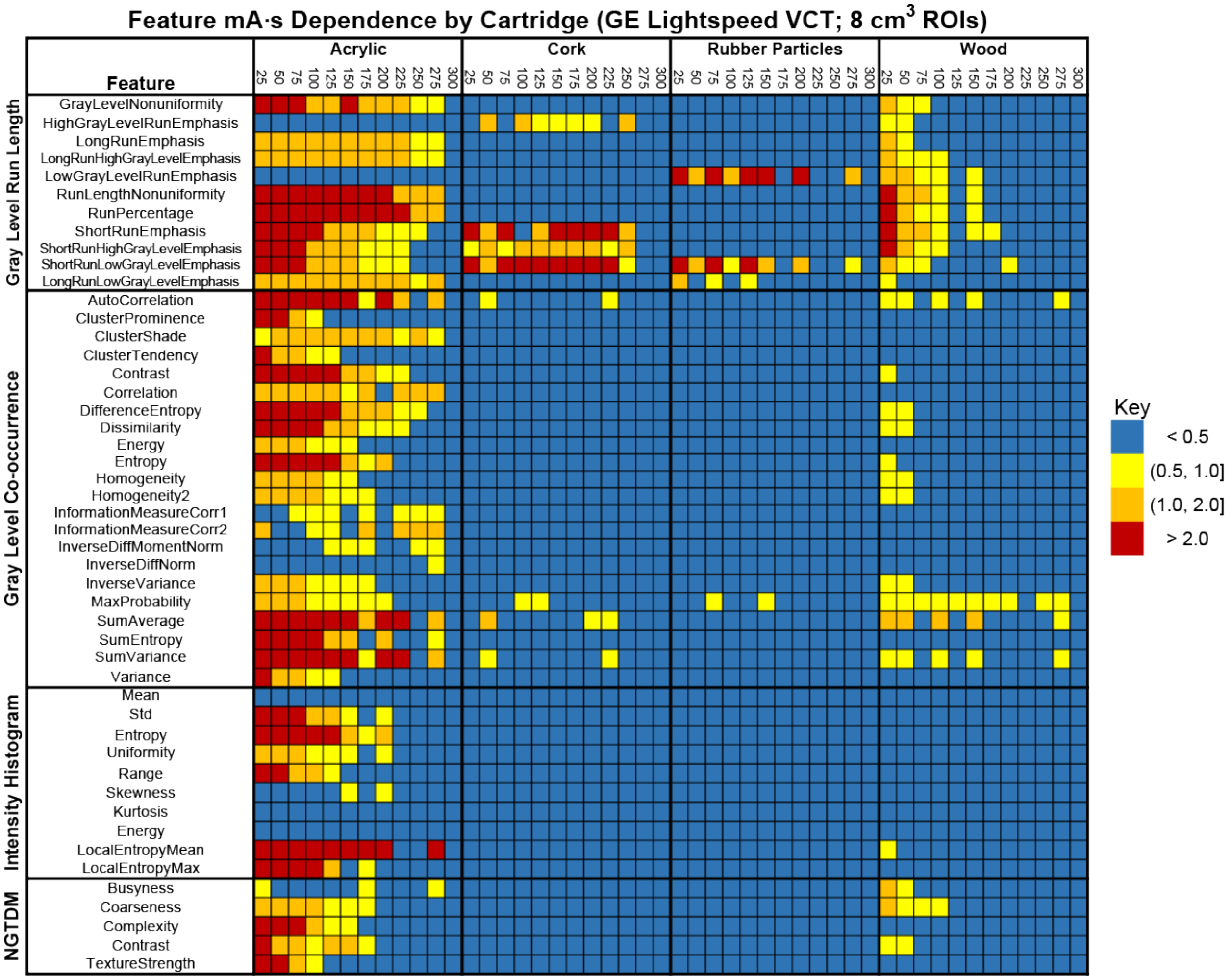

Supplemental Figure 7: Maps of the patient-normalized features extracted from 8 cm^3 ROIs in the acrylic, cork, rubber particle, and sycamore wood cartridges. The columns represent the tube current (mA·s) used to acquire the computed tomography scan and are grouped by the material. Colors other than blue indicate that the effect of the reduced tube current is large relative to the variability of the feature calculated for tumor samples from patients with non-small cell lung cancer. The almost solid blue table for the rubber particle cartridges indicates that reducing the tube current has little effect on the radiomics features. The images were acquired using a GE Lightspeed VCT scanner. GLRL indicates gray-level run length; NGTDM, neighborhood gray tone difference matrix.
